# Lasting effects of prenatal exposure to Cannabis in the retina of the offspring: an experimental study in mice

**DOI:** 10.1186/s40942-021-00314-8

**Published:** 2021-06-30

**Authors:** Paulo Roberto Arruda Zantut, Mariana Matera Veras, Sarah Gomes Menezes Benevenutto, Angélica Mendonça Vaz Safatle, Ricardo Augusto Pecora, Victor Yuji Yariwake, Janaina Iannicelli Torres, Gustavo Sakuno, Marco Antonio Garcia Martins, Aline Adriana Bolzan, Walter Yukihiko Takahashi, Paulo Hilario Nascimento Saldiva, Francisco Max Damico

**Affiliations:** 1 Retina Service, Department of Ophthalmology, University of Sao Paulo Medical School, Sao Paulo, SP Brazil; 2 Laboratory of Experimental Air Pollution, Department of Pathology, University of Sao Paulo Medical School, Sao Paulo, SP Brazil; 3 Ophthalmology Service, Department of Surgery, Veterinary Medicine College and Zootechny, University of Sao Paulo, Sao Paulo, SP Brazil

**Keywords:** Cannabis, Retina, Mice, Prenatal exposure delayed effects, Tomography, Optical coherence, Microscopy

## Abstract

**Background:**

Prenatal exposure to Cannabis is a worldwide growing problem. Although retina is part of the central nervous system, the impact of maternal Cannabis use on the retinal development and its postnatal consequences remains unknown. As the prenatal period is potentially sensitive in the normal development of the retina, we hypothesized that recreational use of Cannabis during pregnancy may alter retina structure in the offspring. To test this, we developed a murine model that mimics human exposure in terms of dose and use.

**Methods:**

Pregnant BalbC mice were exposed daily for 5 min to Cannabis smoke (0.2 g of Cannabis) or filtered air, from gestational day 5 to 18 (N = 10/group). After weaning period, pups were separated and examined weekly. On days 60, 120, 200, and 360 after birth, 10 pups from each group were randomly selected for Spectral Domain Optical Coherence Tomography (SD-OCT) analysis of the retina. All retina layers were measured and inner, outer, and total retina thickness were calculated. Other 37 mice from both groups were sacrificed on days 20, 60, and 360 for retinal stereology (total volume of the retina and volume fraction of each retinal layer) and light microscopy. Means and standard deviations were calculated and MANOVA was performed.

**Results:**

The retina of animals which mother was exposed to Cannabis during gestation was 17% thinner on day 120 (young adult) than controls (P = 0.003) due to 21% thinning of the outer retina (P = 0.001). The offspring of mice from the exposed group presented thickening of the IS/OS in comparison to controls on day 200 (P < 0.001). In the volumetric analyzes by retinal stereology, the exposed mice presented transitory increase of the IS/OS total volume and volume fraction on day 60 (young adult) compared to controls (P = 0.008 and P = 0.035, respectively). On light microscopy, exposed mice presented thickening of the IS/OS on day 360 (adult) compared to controls (P = 0.03).

**Conclusion:**

Gestational exposure to Cannabis smoke may cause structural changes in the retina of the offspring that return to normal on mice adulthood. These experimental evidences suggest that children and young adults whose mothers smoked Cannabis during pregnancy may require earlier and more frequent clinical care than the non-exposed population.

**Supplementary Information:**

The online version contains supplementary material available at 10.1186/s40942-021-00314-8.

## Background

*Cannabis sativa* smoking is a common form of illicit drug dependence in developed and developing countries affecting affects from 2.5% to 4.9% of the world’s population between 15 and 64 years old [1]. In the past 15 years its use has been particularly increasing among women in reproductive age [2, 3]. Cannabis smoking during pregnancy either to treat nausea and vomiting, morning sickness or recreational use is alarmingly increasing, 4% to 7% of women reporting its use at least once [4].

Prenatal exposure to Cannabis smoke is associated to birth weight decrease, higher risk of needing neonatal intensive care, teratogenesis, and cognitive effects [1, 5–12]. Experimental data show that the main psychoactive constituent of Cannabis (Δ9-tetrahydrocannabinol, THC) crosses the placental barrier leading to several adverse effects on the fetus [13]. In addition, THC is present in the breast milk soon after recreational smoking of Cannabis and safe levels of Cannabis use during pregnancy are unknown [14–16].

The endocannabinoid receptors type 1 and 2 are present in all retinal layers [17, 18]. These receptors are related to neural modulation and development within the retina and central nervous system (CNS). Thus, we hypothesize that in utero exposure to Cannabis smoke might interfere with normal retina development.

Research into the impact of Cannabis use during pregnancy has focused primarily on brain development and cognitive outcomes in the offspring. The recent advances in Spectral Domain Optical Coherence Tomography (SD-OCT) imaging allow for the in vivo morphological study through the acquisition of high-definition retinal scans and segmentation of retinal layers. In addition, histologic studies of the retina using stereological techniques allow volumetric analysis of the retinal layers. Therefore, the purpose of this study is to investigate structural changes in the retina of the offspring of pregnant mice exposed to Cannabis smoke during pregnancy with SD-OCT retinal segmentation and histologic stereology analysis.

## Methods

### Animals

All experimental and animal care procedures were in compliance with the National Institutes of Health (NIH) Public Health Service Policy on Humane Care and Use of Laboratory Animals (NIH 2002), adhered to the Association for Research in Vision and Ophthalmology (ARVO) Statement for the Use of Animals in Research, and were approved by the Animal Use Ethics Committee of the University of Sao Paulo Medical School.

Marijuana used in this study received an authorization for scientific purposes by the Núcleo de Perícias Médica Legais—Instituto de Criminalística de Marília legally authorized by the 3^a^ Vara Criminal da Comarca de Marília, Sao Paulo, Brazil. The drug comes from a drug bust conducted by the local police.

Naïve female and male BalbC mice were mated and checked each morning for the presence of a vaginal plug; a positive plug was defined as E0 (E for embryonic period; the letter D will be used in this text to refer to days after birth). Twenty pregnant females were kept in individual cages with food and water ad libitum, in a 12 h:12 h light:dark cycle. Mated females were randomly distributed in Cannabis (CAN) or Filtered Air (FA) groups (n = 10 mice per group). The females from CAN group were exposed to smoke from the burning of 0.2 g of *Cannabis sativa* for five minutes daily inside a smoke apparatus that is described below. Females of the FA group were put into the same apparatus during five minutes daily without burning Cannabis. Exposure to smoke was characterized with presence of THC metabolites in the urine, was checked 24 h after and this procedure was repeated daily from E5 until E17.

Animals gave birth between E19 and E2. After weaning period (P21), male and female offspring were allocated separately in cages (3–4 animal/cage) according to gestational treatment. In this study, SD-OCT imaging data of 10 pups randomly assigned to each group were prospectively collected on D60, D120, D200, and D360. Other 37 pups (18 from CAN group and 19 from FA group) were selected for histologic analysis and were euthanized by overdose of isoflurane (Forane, Abbott Laboratories Argentina S.A., Buenos Aires, Argentina) on D20, D60, and D360.

### Smoke apparatus

The smoke-generating and inhalation device was developed by the Experimental Atmospheric Pollution Lab at University of Sao Paulo as described elsewhere [19]. Briefly, it consisted of three separated chambers for burning, mixing and exposition. Each pregnant mouse spent 5 min daily inside de exposition chamber for inhalation of the burn of *Cannabis sativa* 0.2 g. The cigarettes were prepared with commercially available paper wrappings.

### Spectral domain optical coherence tomography imaging

Subcutaneous injection of a mixture containing 67 mg/kg ketamine and 12 mg/kg xylazine was used for mice anesthesia. Pupils were dilated using tropicamide 1% eye drops (Ciclomidrin, Latinofarma, Sao Paulo, Brasil). Commercially available lubricants were used to prevent corneal drying (Systane, Alcon, Sao Paulo, Brazil).

SD-OCT images were acquired with the Heidelberg Retina Angiograph (HRA) Spectralis (Heidelberg Engineering, Germany). Three horizontal SD-OCT linear B-scans, centered on the optic nerve head, were obtained from each eye. SD-OCT scans were acquired in the automatic real-time mode (ART), averaging 30 frames per image, with a 30º covering field. The images with better quality were selected for analysis (Additional file 1). Retinal layer segmentation was performed automatically with the device internal software and manually corrected when necessary. The retina was segmented in: NFL + GCL (nerve fiber layer plus ganglion cell layer), IPL (inner plexiform layer), INL (inner nuclear layer), OPL (outer plexiform layer), ONL (outer nuclear layer), IS/OS (photoreceptors inner segments and outer segments junction), and RPE (retinal pigment epithelium). Inner retina included NFL + GCL, IPL, and INL and outer retina thickness included OPL, ONL, IS/OS, and RPE (Additional file 1). Thickness measurements were performed 600 µm from the optic disc margin, nasally and temporally. The average values of total retina, inner, outer retina, and from each segmented layer thicknesses were used for analysis.

### Retinal stereology and light microscopy

All tissue processing techniques were performed as described previously [21, 22]. Briefly, enucleated eyes were fixed in buffered 4% paraformaldehyde and transferred after 24 h to a 70% alcohol solution for a week. Then, eyes were included in 14% bacteriological agar blocks to guarantee the orientation and processed routinely for histology (paraffin). All blocks were serially sectioned at 5 µm (parallel to optic nerve axis) and at 200 µm intervals one section was collected into glass slides for stereological estimation of volumes (Additional file 2: Figure 2A). All slides were scanned and photographed using *Panoramic viewer* (3DHISTECH Ltd., Budapest, Hungary). *ImageJ* (National Institutes of Health, Bethesda, MD) software were used to analyze and generate the test systems for stereology. Nearly 24 images per eye were used and a point test system with a reference area of 17 × 10^3^ µm^2^ per point were superimposed to estimate the volumes by the Cavalieri’s Principle for volumetric measurements of the retinal layers (Additional file 2: Figure 2B). The total volume of the retina and the volume fraction of each retinal layer were calculated. The volume fraction expresses the proportion of a single layer within the whole retina.

For retinal layers thickness measurements, randomly selected cuts from each retina were directly measured at 15 to 20 different points, from which an average thickness value for each retinal layer was obtained (Additional file 3).

### Statistical analysis

The Statistical Package for Social Sciences (SPSS) software version 17.0 was used for Statistical analysis. Collected data from SD-OCT imaging and stereology were analyzed by three-way ANOVA followed by multivariate ANOVA (MANOVA) for dependent variables testing (age and sex of the offspring, and mother exposition to either Cannabis smoke or FA during pregnancy). Data are presented as mean ± standard deviation (SD). The null hypothesis was rejected at probability level of P < 0.05.

## Results

### Retinal thickness under SD-OCT

**Fig. 1:**
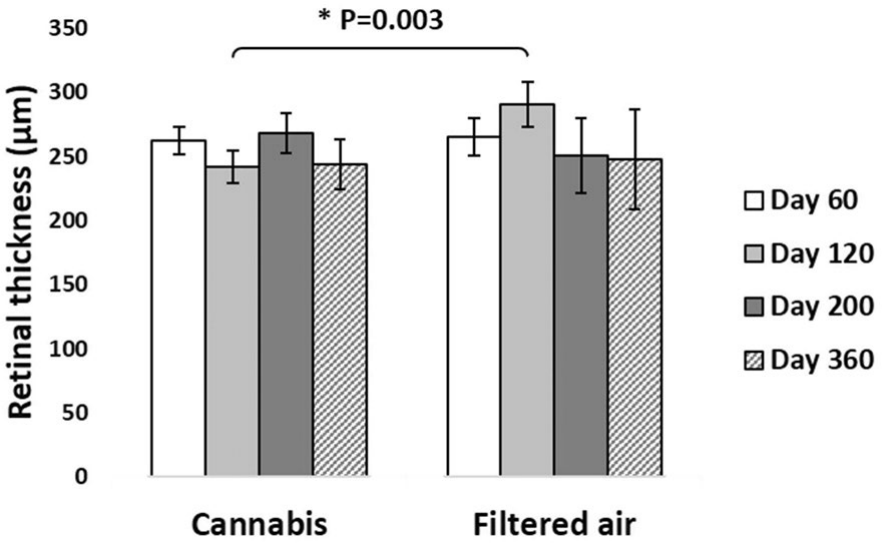
Total retina thickness on SD-OCT of the offspring of pregnant mice exposed to Cannabis smoke or filtered air during pregnancy (mean ± standard deviation). *P = 0.003 (MANOVA, 95% CI: 251–263, age*exposition)

On D120, **total retina** of the offspring of the CAN group was 17% thinner than in FA group (242 ± 13 µm vs. 291 ± 17 µm, respectively). In multivariate regression analysis, age*exposition was statistically significant for this outcome (95% CI: 251–263, P = 0.003; Fig. 1).

**Fig. 2:**
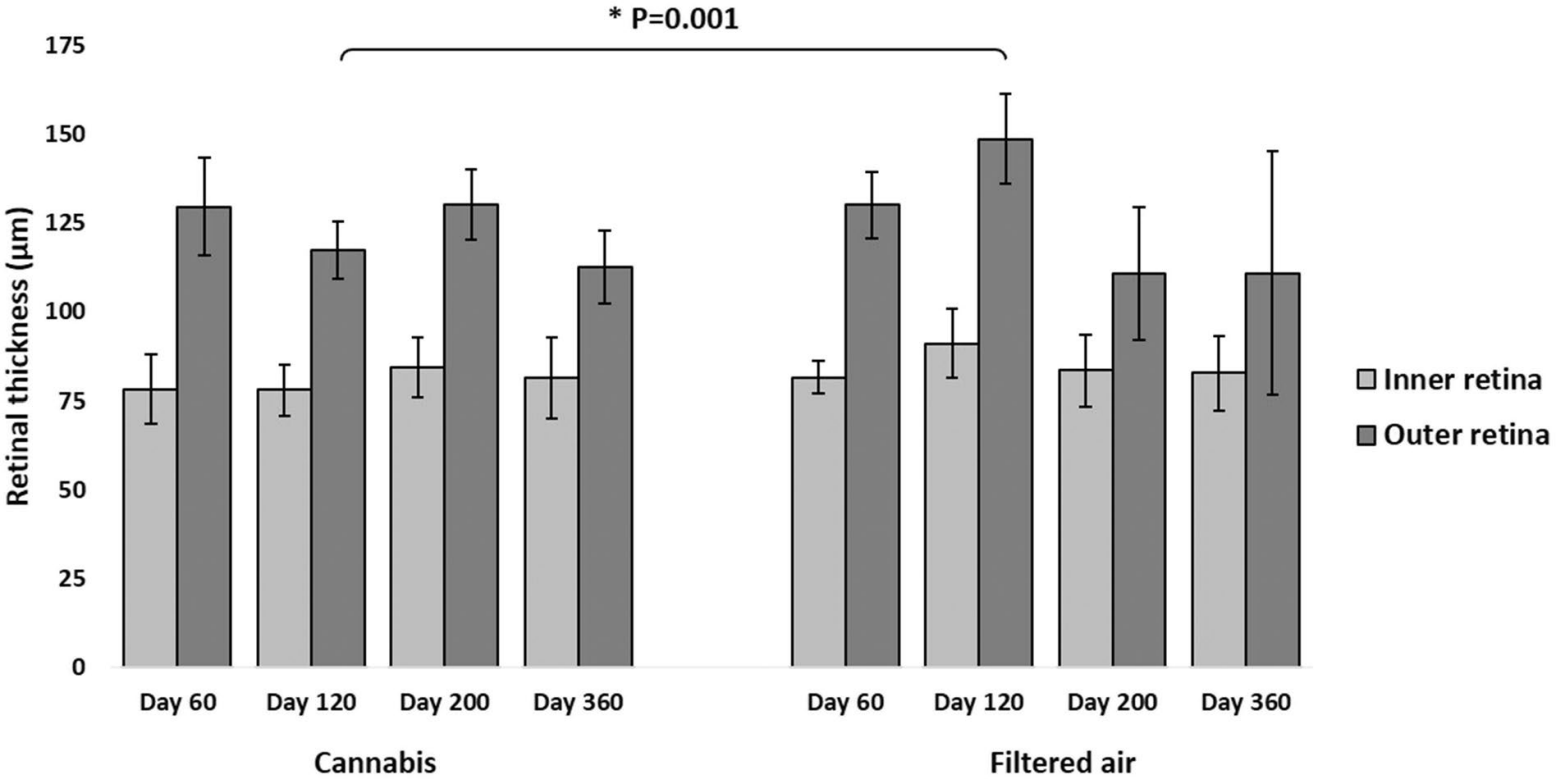
Inner and outer retina thickness on SD-OCT of the offspring of pregnant mice exposed to Cannabis smoke or filtered air during pregnancy (mean ± standard deviation). Inner retina included nerve fiber layer, ganglion cell layer (NFL + GCL), inner plexiform layer (IPL), and inner nuclear layer (INL). Outer retina included outer plexiform layer (OPL), outer nuclear layer (ONL), photoreceptors inner segments and outer segments junction (IS/OS), and retinal pigment epithelium (RPE). *P = 0.001 (MANOVA, 95% CI: 117–126, age*exposition)

On the same time point (D120), **outer retina** of the offspring was 21% thinner in CAN group than in FA group (117 ± 8 µm vs. 149 ± 13 µm, respectively). Multivariate regression analysis showed the influence of age*exposition over these outcomes (95% CI: 117–126, P = 0.001; Fig. 2).

There was no difference between groups regarding inner retina thickness.

**Fig. 3:**
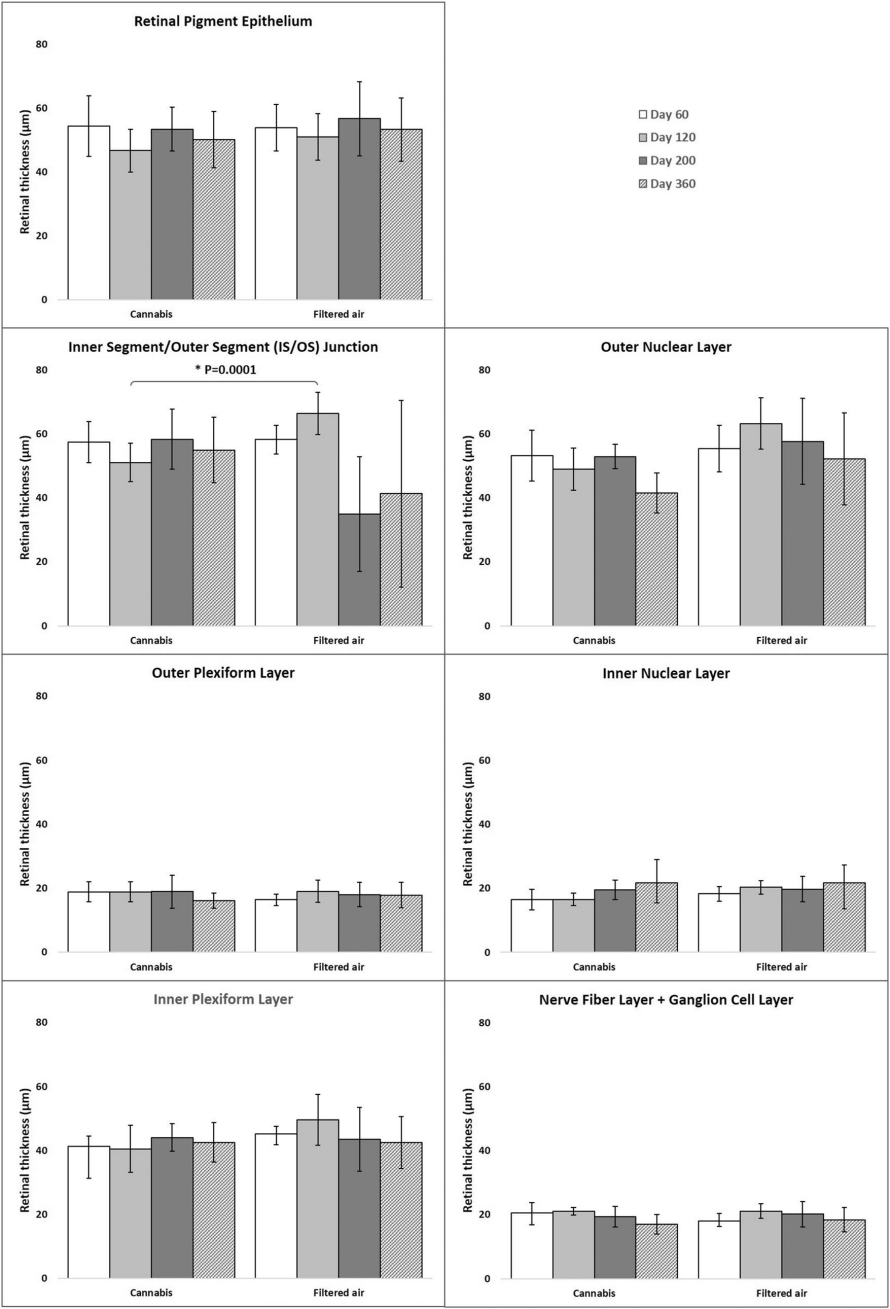
Retinal layers thickness on SD-OCT of the offspring of pregnant mice exposed to Cannabis smoke or filtered air during pregnancy (mean ± standard deviation).*P = 0.0001 (MANOVA, 95% CI: 47–54, age*exposition)

The analysis of the thickness of each retinal layer of the offspring showed that on D200 the **IS/OS** from the CAN group was 68% thicker than in the FA group (58 ± 9 µm vs. 35 ± 18 µm, respectively). Results were influenced by age*exposition (95% CI: 47–54; P = 0.0001; Fig. 3).

In multivariate regression analysis, after controlling for age, sex, and exposition, NFL + GCL, IPL, INL, OPL, ONL, and RPE thicknesses were similar in CAN and FA groups.

### Volumetric analysis under retinal stereology

In multivariate regression analysis, after controlling for age, sex, and exposition, the only retinal layers of the offspring that presented total volume and volume fraction differences were IS/OS and ONL.

**Fig. 4:**
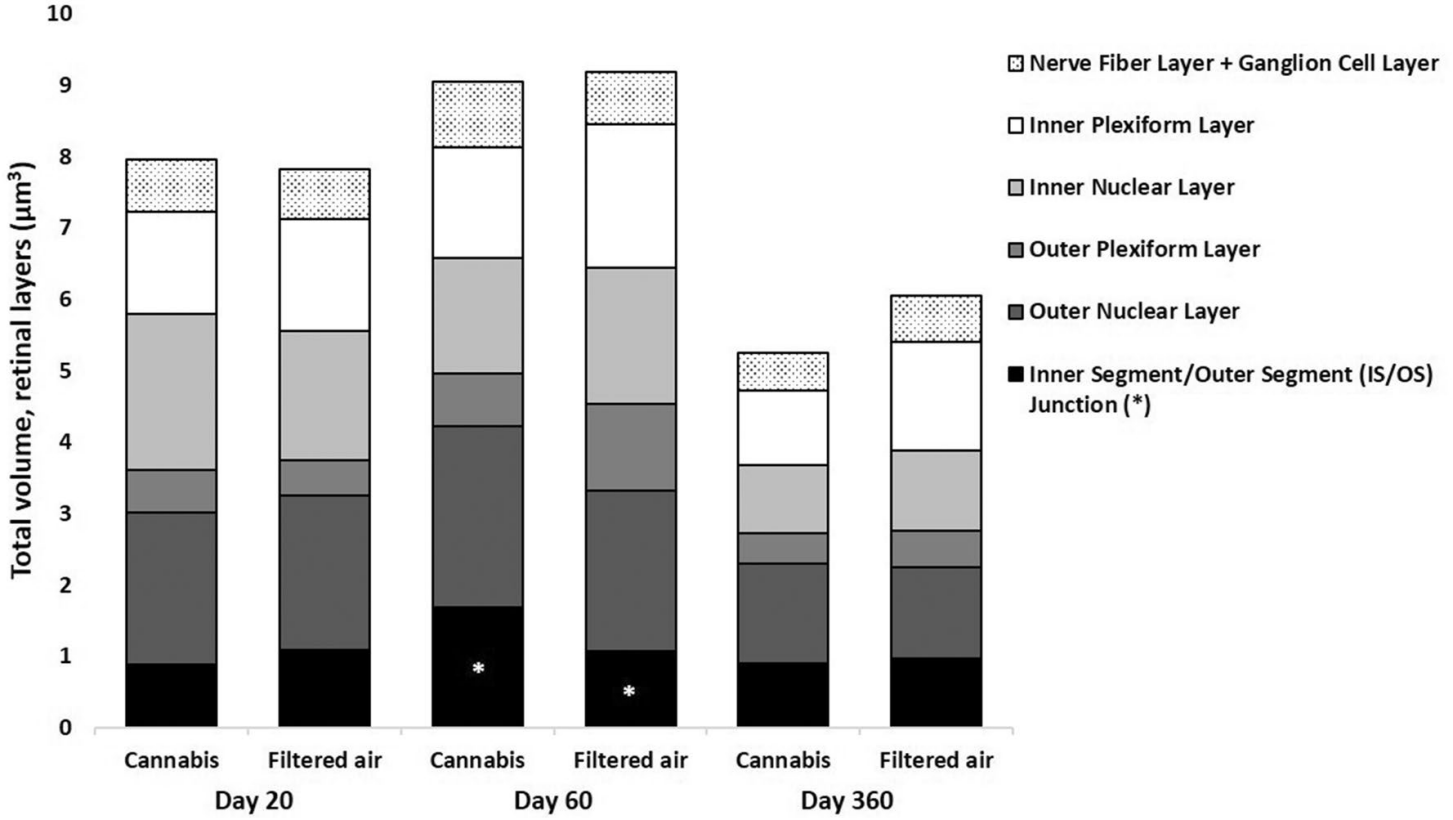
Mean total volume of each retina layer under stereology of the offspring of pregnant mice exposed to Cannabis smoke or filtered air during pregnancy. *P = 0.008 (MANOVA, 95% CI: 0.8–1.5, age*exposition)

The **total volume of the IS/OS** in the CAN group on D20, D60, and D360 was 0.9 ± 0.3 µm^3^, 1.7 ± 0.5 µm^3^, and 0.9 ± 0.3 µm^3^, respectively, and in the FA group was 1.1 ± 0.3 µm^3^, 1.1 ± 0.6 µm^3^, and 1.0 ± 0.3 µm^3^, respectively. Results were influenced by age*exposition (95% CI: 0.8–1.5, P = 0.008; Fig. 4).

In multivariate regression analysis, after controlling for age, sex, and exposition, the volume of the total retina and each other retinal layers were similar in CAN and FA groups.

**Fig. 5:**
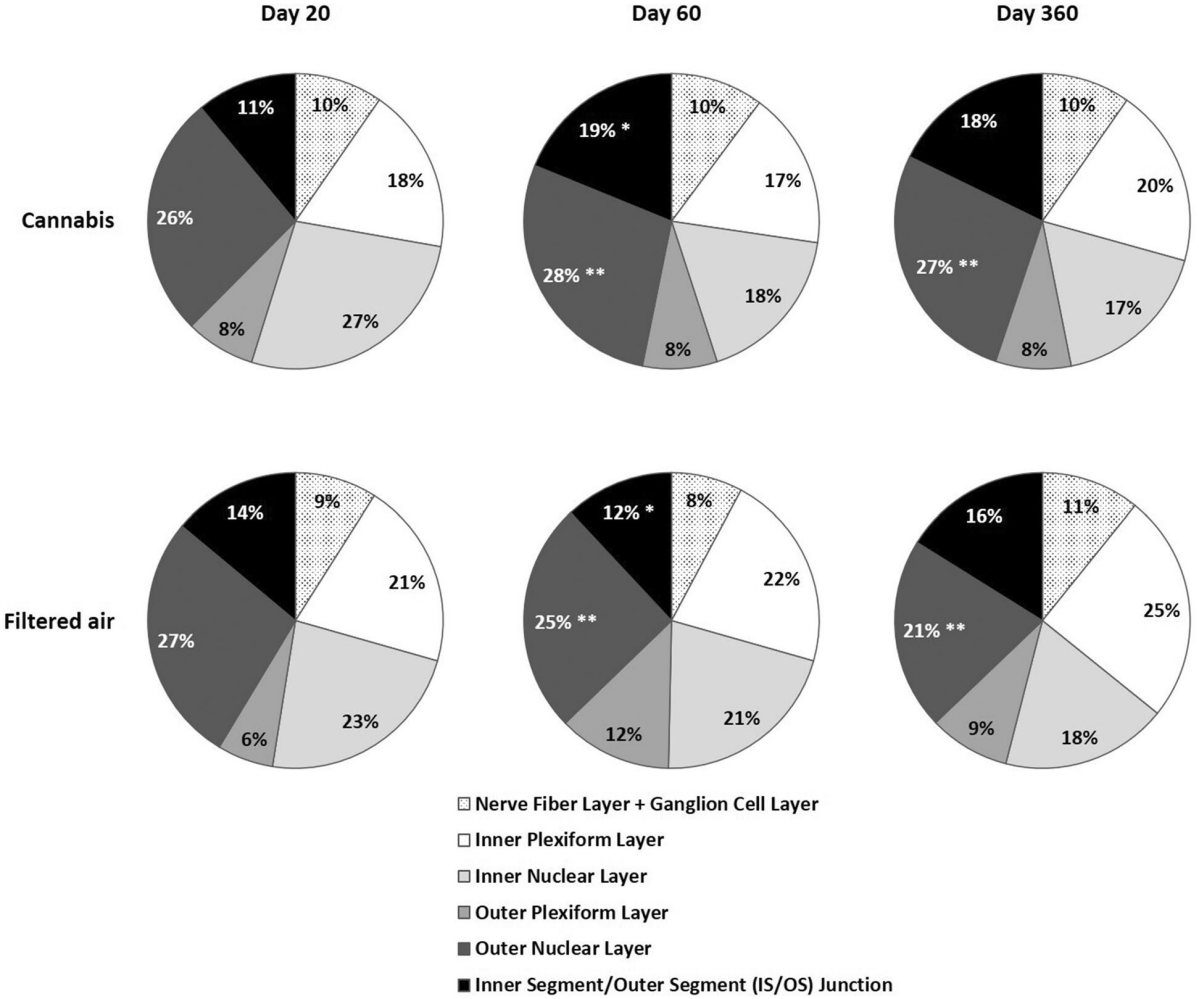
Mean volume fraction of each retina layer under stereology of the offspring of pregnant mice exposed to Cannabis smoke or filtered air during pregnancy. *P = 0.035 (MANOVA, 95% CI: 10.6–17.4, age*exposition) **P = 0.005 (MANOVA, 95% CI: 22.8–26.4, sex*exposition)

The **volume fraction of the IS/OS** in the CAN group on D20, D60, and D360 was 11 ± 4%, 19 ± 5%, and 18 ± 7%, respectively, and in the FA group was 14 ± 4%, 12 ± 7%, and 16 ± 4%, respectively. Results were influenced by age*exposition (95% CI: 10.6–17.4, P = 0.035; Fig. 5).

The **volume fraction of the ONL** in the CAN group on D20, D60, and D360 was 26 ± 4%, 28 ± 4%, and 27 ± 4%, respectively, and in the FA group was 27 ± 45, 25 ± 9%, and 21 ± 4%, respectively. Results were influenced by sex*exposition (95% CI: 22.8–26.4, P = 0.005; Fig. 5).

In multivariate regression analysis, after controlling for age, sex, and exposition, the volume fractions of the other retinal layers were similar in CAN and FA groups.

### Retinal morphology and thickness under light microscopy

**Fig. 6:**
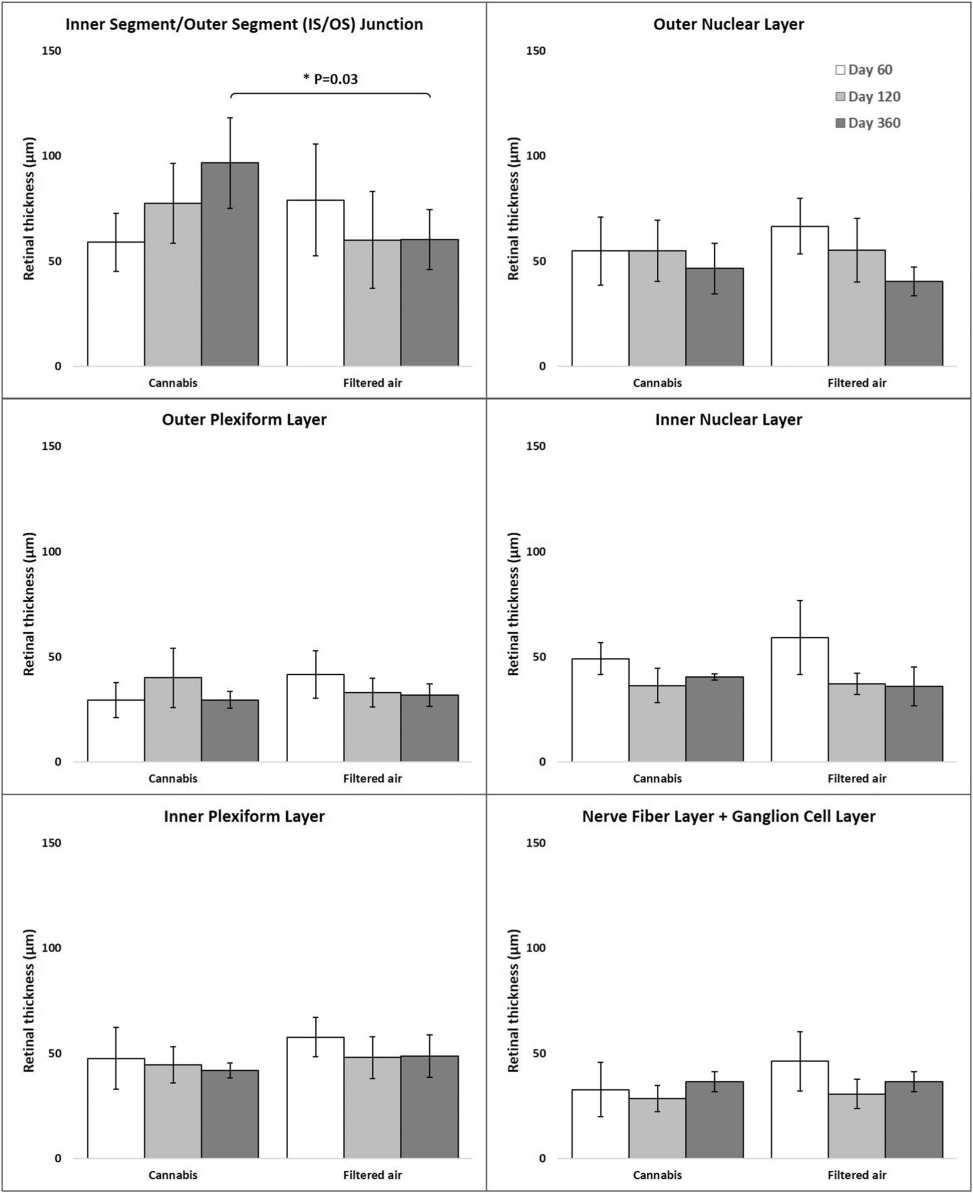
Retina layers thickness under light microscopy of the offspring of pregnant mice exposed to Cannabis smoke or filtered air during pregnancy (mean ± standard deviation). *P = 0.03 (MANOVA, 95% CI: 55–78, age*exposition)

The **IS/OS thickness** in the CAN group on D20, D60, and D360 was 59 ± 14 µm^3^, 77 ± 19 µm^3^, and 97 ± 21 µm^3^, respectively, and in the FA group was 74 ± 26 µm^3^, 60 ± 23 µm^3^, and 60 ± 14 µm^3^, respectively. Results were influenced by age*exposition (95% CI: 55–78, P = 0.03; Fig. 6).

In multivariate regression analysis, after controlling for age, sex, and exposition, there was no difference on either the thickness of total retina or any other retinal layers between CAN and FA groups.

## Discussion

In this study we investigated the structural changes in the retina of the offspring of pregnant mice exposed to Cannabis smoke during pregnancy. Our results show that mother exposure to Cannabis smoke during pregnancy causes morphological retinal changes in the offspring that are transitory and return to normal by adulthood. The offspring presents retinal thinning on D120 (young adult) due to outer retina thinning but it returns to normal values on D360 (adult mice). There is also a transitory thickening of the IS/OS from D60 to D200.

To the best of our knowledge, this is the first experimental study to evaluate the lasting effects of gestational exposure to Cannabis smoke on the retinal structure of the offspring. The method of exposition to Cannabis smoke used in our study mimics real life Cannabis use in terms of dose and use. The dosage to which the subjects were exposed was comparable to those found in humans on recreational Cannabis smoking [19]. The offspring was followed from birth up to one year old (adulthood) so we were able to detect early and late morphological retinal effects in animals whose mothers were exposed to Cannabis smoke during pregnancy.

The SD-OCT measurements showed three significant findings in the offspring of mice exposed to Cannabis smoke during pregnancy compared to controls. First, total retina was 17% thinner on D120 (young adult mice). Second, total retinal was thinner because of the 21% outer retina thinning that occurred on the same time point. And third, the IS/OS was thicker on D200 compared to controls (young adult mice). Eventually, retinal thickness returned to normal values on D360 (adult mice). These findings suggest that Cannabis use during pregnancy causes reversible morphological damage to photoreceptors of the offspring.

The IS/OS thickening seen on D200 may be secondary to the effects of Cannabis on remodeling, apoptosis, and neuroprotection. It is known that the endocannabinoid system plays an important role in neuroprotection due to the promotion of cell survival and inhibition of apoptosis. These mechanisms are very important during the development of the retina, when connections between neurons are in progress [20]. Thus, our results may represent an attempt to decrease cellular losses by apoptosis as studies have demonstrated the role of the endocannabinoid system on neuroplasticity and its neuroprotective action in traumatic and ischemic injuries, inflammation, and neurological damage to the CNS [20]. However, the IS/OS thickening on SD-OCT is transitory and it matches our results on retinal stereology and light microscopy. Although retinal stereology and light microscopy were not performed on D120 and D200, retinal stereology showed that total volume and volume fraction of the IS/OS increased during young adulthood (D60) and returned to normal on adulthood (D360). In contrast, IS/OS thickness did not return to normal values on D360 under light microscopy.

In addition to changes in the IS/OS, retinal stereology showed increase of ONL volume fraction in the young adult mice (D60), suggesting that the same process that occurs in the inner and outer segments of the photoreceptors may also occur in the cell bodies of the photoreceptors. A case report showed reversible a-wave amplitude decrease in the ERG after Cannabis smoking for all scotopic responses, suggesting that photoreceptors function is affected by Cannabis inhalation [21].

Other studies report signs of neuroretinal damage secondary to Cannabis abuse in adult humans. One case report described OCT imaging of subretinal blebs following vision loss in a strong hashish user [22]. González-Pérez et al. found electroretinographic signal reduction in a cohort of Cannabis users, also suggesting photoreceptor damage [23]. Electroretinographic changes were also suggested by further studies in Cannabis users [21, 24]. Bipolar and ganglion cell dysfunction have been also reported in a flash and pattern electroretinogram case–control study suggesting the neurotoxic effects of Cannabis use impair retinal processing [24]. Several other studies also suggest that prenatal exposure to Cannabis may cause ocular damage by other mechanisms, such as vascular or teratogenic [25–27].

Clinical studies have shown that the integrity of the retinal layers on SD-OCT is associated with visual acuity and retinal sensitivity [28, 29]. An experimental study using adult BALB/c mice showed that 2-month treatment with intraperitoneal injection of THC caused functional retinal impairment and increased apoptosis in photoreceptors by induction of inflammatory response and oxidative stress. Although the via of administration of THC was different from our study and retinal damage was detected directly in the exposed mice, these are evidences that the use of Cannabis induces an inflammatory state in the retina and it might be related to morphological changes [30]. In our study it is not possible to correlate the morphological changes in young adult and adult mice with functional retinal changes as the design of the study does not allow conclusions to be drawn in this regard. It remains unknown whether Cannabis use causes morphological, neuroretinal disfunction or both [31, 32].

The effects of the active component of Cannabis (THC) can be detected in almost all organs and is mediated by CB1 and CB2 receptors. A study on the normal embryonic development of rats retina shows that ganglion cells and the INL of the retina express CB1 receptors as early as the E15 and E20 [33]. The endocannabinoid system plays an important physiological neuroprotective role in the nervous system favoring cell survival [34], plasticity, and maturation [35, 36]. During the development of the retina there are critical stages of cell elimination (apoptosis), sometimes to remove cells that have not established communications or compromised cells [37–39]. Our results suggest that prenatal inhalation of Cannabis smoke by pregnant mice interferes with retinal development of the offspring. This is the experimental confirmation of an expected finding as the endocannabinoid system is present in all retinal layers and in other CNS areas [40].

In this study, we also examined the potential effect of sex differences in gestational Cannabis exposure on retina’s development. Sex-dependent differences have been frequently observed in the biological and behavioral effects of Cannabis use in both animal and human studies [41–45]. Cannabinoids have been shown to exert sex-dependent effects also in other aspects, such as energy balance, anxiety and depression [46]. The pre-natal stress activates gonadal hormones and the hypothalamic–pituitary–adrenal system and it causes changes in the neurotransmission modulation and sex hormones [47]. The density and function of CB1 receptor has also been linked to sex differences in behavioral and predisposing to drug addiction in rats [42].

Our study has some limitations. Despite similarities in retinal layers and degree of vascularization [48, 49], human and mice eyes have very different eye volumes. In addition, macula is absent in the murine model and there may be drug susceptibility differences among those species [50–52]. The macula is an important anatomic landmark for SD-OCT scans. To overcome the absence of macula in the murine model we used the optic nerve as reference for the SD-OCT scans and measurements were made in triplicates.

Other limitations are the small volume of urine available for the quantification of THC metabolites and the low dose of THC in the Cannabis used in this study. Although we checked daily for the concentration of carboxi-THC in the urine of the pregnant mice, the Cannabis used in this study had significantly less THC than the used nowadays. Therefore, the precise determination of the dose of exposition has been hampered. However, our exposition was very similar to the conducted by Lichtman et al.[52] and the concentration of carboxi-THC detected in our urine samples was the same as reported by the authors for the burning of the same amount used in this study (0.2 g of *Cannabis sativa*, 5.6 mg/kg). Finally, the relatively low concentration of THC in the Cannabis used in this study values our findings and reinforces the potential toxic effects of Cannabis in the retina of the offspring of pregnant mice exposed to Cannabis smoke.

In terms of Public Health, our findings are particularly relevant as there is an increasing worldwide interest in the legalization of Cannabis. Lately, important studies have been showing positive effects of Cannabis therapeutic use in scenarios of untreatable pain, terminal cancer, obesity, and glaucoma [53]. During pregnancy, Cannabis smoking is commonly used to treat nausea and vomiting, morning sickness or recreational use. However, little is known on the effects of Cannabis in organs formation, including the eye.

In summary, we have shown for the first time that gestational exposure to Cannabis smoke may cause structural changes in the retina of the offspring that return to normal on mice adulthood. These are experimental evidences that suggest that children and young adults whose mothers smoked Cannabis during pregnancy may require earlier and more frequently clinical care than the non-exposed population.

## Supplementary Information

**Additional file 1: Figure S1** Retinal segmentation in a BALB/c mouse SD-OCT B-scan.

**Additional file 2: Figure S2** Stereological analysis of the retina.

**Additional file 3: Figure S3:** Photomicrographs of histological cross sections of the mouse retina.

## Data Availability

The datasets used and/or analysed during the current study are available from the corresponding author on reasonable request.

## Abbreviations

THC: Δ9-Tetrahydrocannabinol
CNS: Central nervous system
SD-OCT: Spectral Domain Optical Coherence Tomography
NIH: National Institutes of Health
ARVO: Association for Research in Vision and Ophthalmology
E: Embryonic period
D: Days after birth
CAN: Cannabis group
FA: Filtered air group
HRA: Heidelberg Retina Angiograph
ART: Automatic real-time mode
NFL + GCL: Nerve fiber layer plus ganglion cell layer
IPL: Inner plexiform layer
INL: Inner nuclear layer
OPL: Outer plexiform layer
ONL: Outer nuclear layer
IS/OS: Photoreceptors inner segments and outer segments junction
RPE: Retinal pigment epithelium
SPSS: Statistical Package for Social Sciences
ANOVA: Analysis of variance
MANOVA: Multivariate analysis of variance
SD: Standard deviation
CI: Confidence interval
OCT: Optical Coherence Tomography
CB1: Cannabinoid receptor 1
CB2: Cannabinoid receptor 2
