# Supplementary material for: Lasting effects of prenatal exposure to Cannabis in the retina of the offspring: an experimental study in mice
Source: Int J Retina Vitreous. 2021 Jun 30;7:45. doi: 10.1186/s40942-021-00314-8 (PMC8246684; doi:10.1186/s40942-021-00314-8)
Supplement: Supplementary file 1 — Additional file 1: Figure S1 Retinal segmentation in a BALB/c mouse SD-OCT B-scan. [file 40942_2021_314_MOESM1_ESM.docx]

**
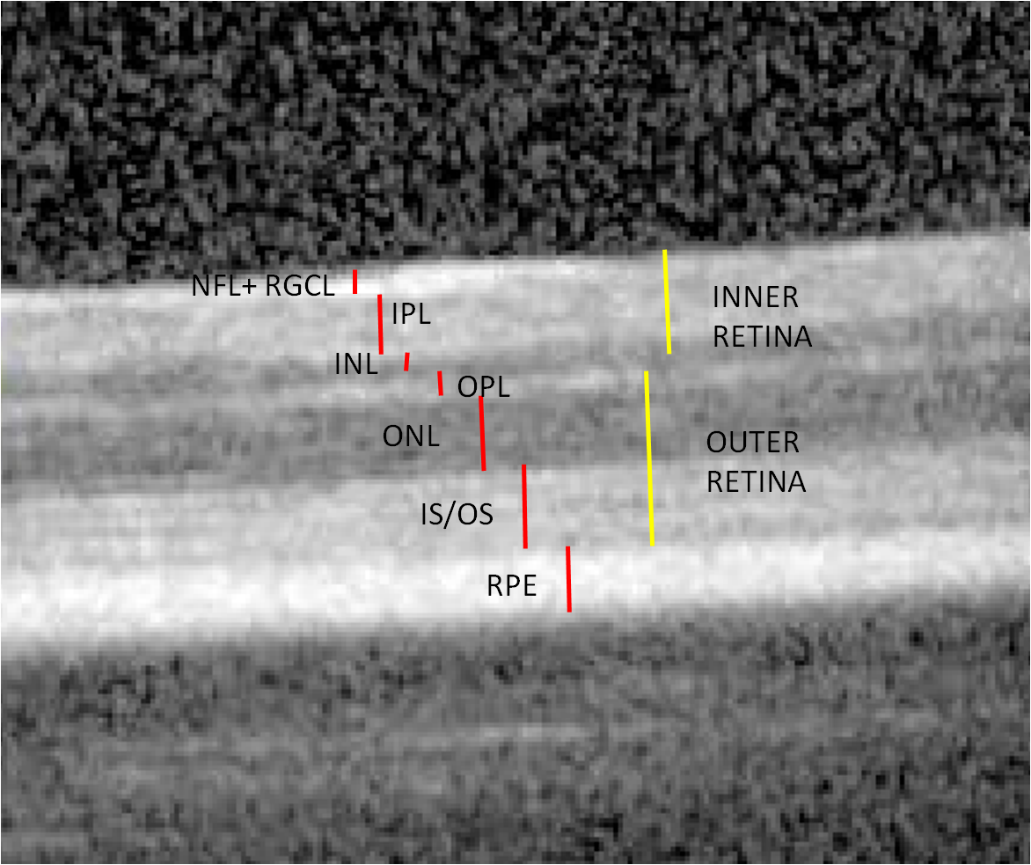
**

**Supplementary File 1:** Retinal segmentation in a BALB/c mouse SD-OCT B-scan. NFL+RGCL: Nerve Fiber Layer-Retinal Ganglion Cell Layer; IPL: Inner Plexiform Layer; INL: Inner Nuclear Layer; OPL: Outer Plexiform Layer; ONL: Outer Nuclear Layer; IS-OS: Inner Boundary of Inner Segment-Outer Segment; RPE: Retinal Pigment Epithelium
