# Supplementary material for: Lasting effects of prenatal exposure to Cannabis in the retina of the offspring: an experimental study in mice
Source: Int J Retina Vitreous. 2021 Jun 30;7:45. doi: 10.1186/s40942-021-00314-8 (PMC8246684; doi:10.1186/s40942-021-00314-8)
Supplement: Supplementary file 2 — Additional file 2: Figure S2 Stereological analysis of the retina. [file 40942_2021_314_MOESM2_ESM.docx]

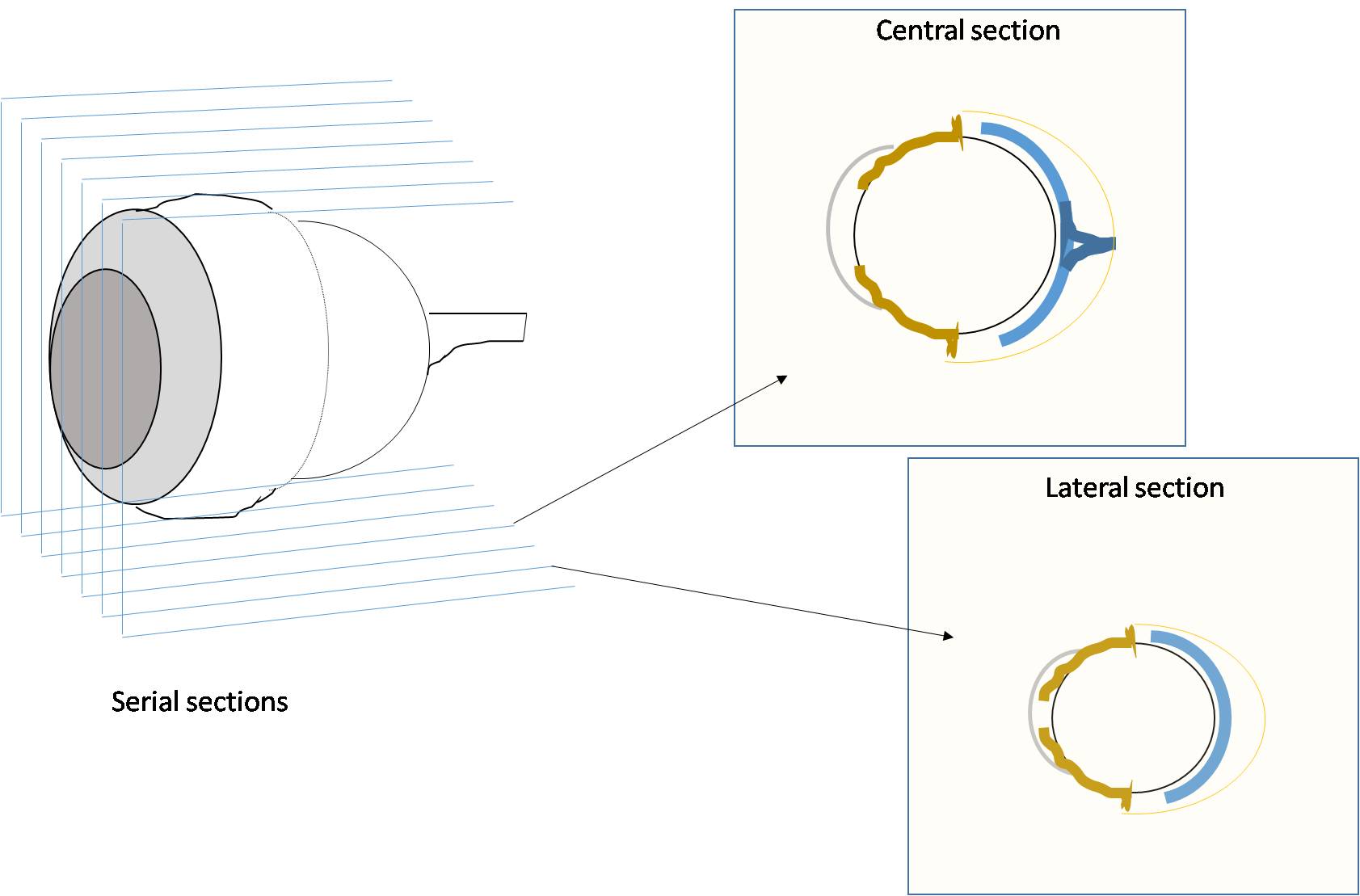


**A**

**B**


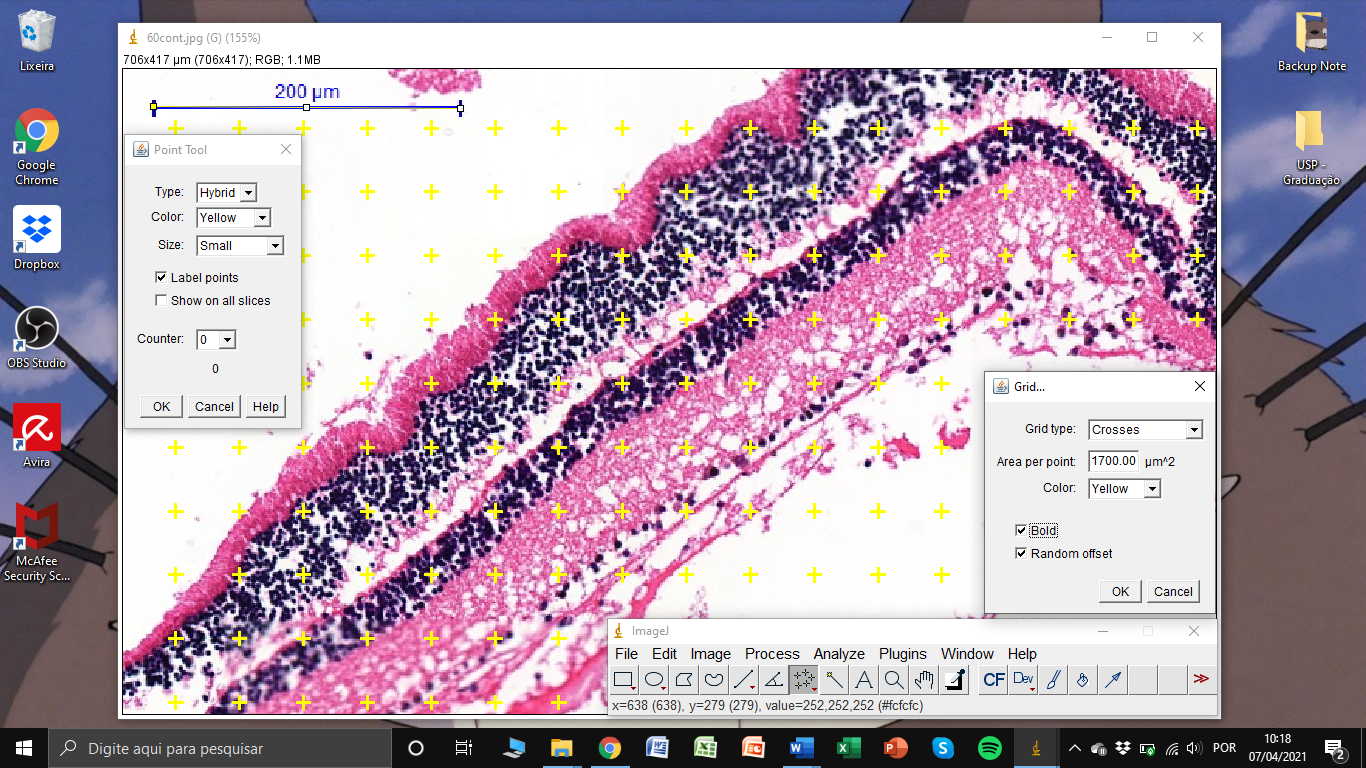


**B**

**Supplementary File 2:** Stereological analysis of the retina. (A) Representation of serial sections of the eye collected for stereological analysis. (B) Application of Cavalieri’s Principle for the volume analysis of retinal layers using the software *ImageJ*.
