# Supplementary material for: Lasting effects of prenatal exposure to Cannabis in the retina of the offspring: an experimental study in mice
Source: Int J Retina Vitreous. 2021 Jun 30;7:45. doi: 10.1186/s40942-021-00314-8 (PMC8246684; doi:10.1186/s40942-021-00314-8)
Supplement: Supplementary file 3 — Additional file 3: Figure S3: Photomicrographs of histological cross sections of the mouse retina. [file 40942_2021_314_MOESM3_ESM.docx]

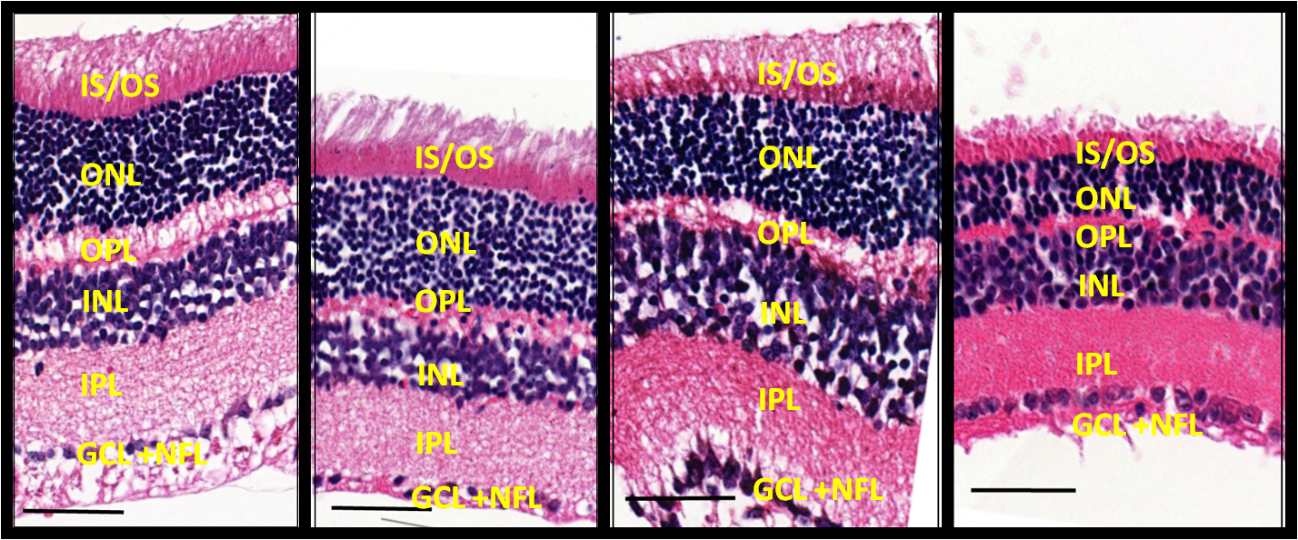


**A**

**D**

**C**

**B**

**Supplementary File 3:** Photomicrographs of histological cross sections of the mouse retina. (A) 60 days – control; (B) 360 days – control; (C) 60 days – Cannabis; (D) 360 days – Cannabis. GCL+NFL: Ganglion Cell Layer + Nerve Fiber Layer; IPL: Inner Plexiform Layer; INL: Inner Nuclear Layer; OPL: Outer Plexiform Layer; ONL: Outer Nuclear Layer; IS-OS: Inner Boundary of Inner Segment-Outer Segment. Bars = 50 µm.
